# Biosynthesis of eriodictyol from tyrosine by *Corynebacterium glutamicum*

**DOI:** 10.1186/s12934-022-01815-3

**Published:** 2022-05-14

**Authors:** Xia Wu, Jingyi Liu, Dan Liu, Miaomiao Yuwen, Mattheos A. G. Koffas, Jian Zha

**Affiliations:** 1 School of Food and Biological Engineering, Shaanxi University of Science and Technology, Xi’an, 710021 Shaanxi China; 2 Department of Chemical and Biological Engineering, Center for Biotechnology and Interdisciplinary Studies, Rensselaer Polytechnic Institute, Troy, NY 12180 USA

**Keywords:** Eriodictyol, *Corynebacterium glutamicum*, Naringenin, Flavonoid, Tyrosine

## Abstract

**Background:**

Eriodictyol is a bioactive flavonoid compound that shows potential applications in medicine development and food processing. Microbial synthesis of eriodictyol has been attracting increasing attention due to several benefits. In this study, we employed a GRAS strain *Corynebacterium glutamicum* as the host to produce eriodictyol directly from tyrosine.

**Results:**

We firstly optimized the biosynthetic module of naringenin, the upstream intermediate for eriodictyol production, through screening of different gene orthologues. Next, to improve the level of the precursor malonyl-CoA necessary for naringenin production, we introduced *matB* and *matC* from *Rhizobium trifolii* into *C. glutamicum* to convert extracellular malonate to intracellular malonyl-CoA. This combinatorial engineering resulted in around 35-fold increase in naringenin production from tyrosine compared to the initial recombinant *C. glutamicum*. Subsequently, the *hpaBC* genes from *E. coli* encoding 4-hydroxyphenylacetate 3-hydroxylase were expressed in *C. glutamicum* to synthesize eriodictyol from naringenin. Further optimization of the biotransformation process parameters led to the production of 14.10 mg/L eriodictyol.

**Conclusions:**

The biosynthesis of the *ortho*-hydroxylated flavonoid eriodictyol in *C. glutamicum* was achieved for the first time via functional expression of *E. coli hpaBC*, providing a baseline strain for biosynthesis of other complex flavonoids. Our study demonstrates the potential application of *C. glutamicum* as a host microbe for the biosynthesis of value-added natural compounds from tyrosine.

## Background

Eriodictyol is a hydroxylated flavonoid compound widely present in citrus fruits, vegetables, and many medicinal plants [1]. This plant-based natural product has antioxidative, anti-inflammatory and neuroprotective effects, and can thus be potentially used in pharmaceutical, nutraceutical, or food industries [1–3]. At present, the main way of obtaining eriodictyol is via extraction from plants or chemical synthesis [4]. As an alternative, microbial production of eriodictyol using engineered microorganisms is increasingly becoming an attractive approach due to its various advantages such as independence of seasonal variations, purity of end products, and rapid production [5, 6].

**Fig. 1:**
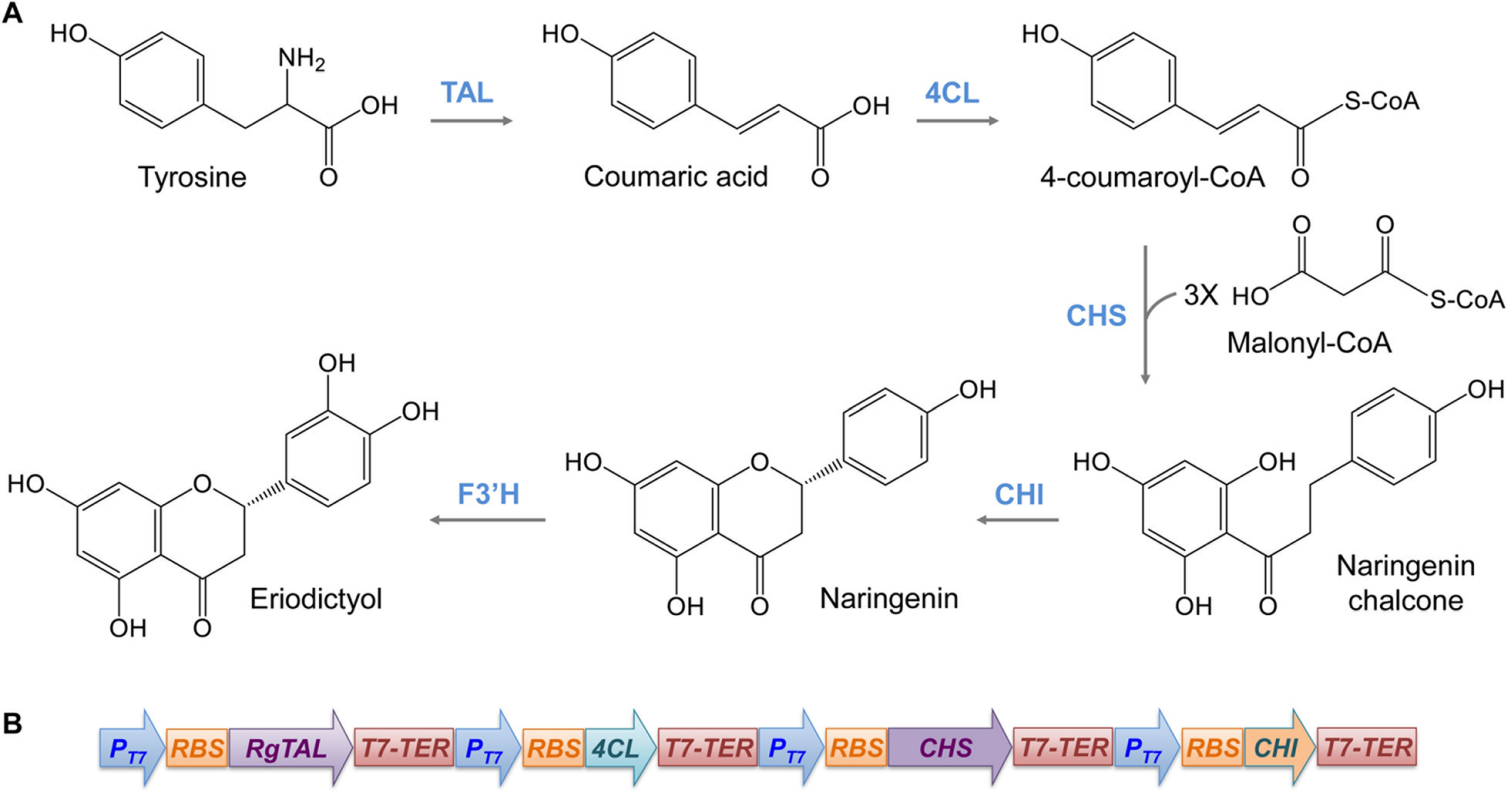
Biosynthetic pathway of eriodictyol from tyrosine. **A** The pathway introduced into the recombinant *C. glutamicum* in this study. **B** The monocistronic arrangement of the pathway genes responsible for naringenin production from tyrosine. TAL tyrosine ammonia lyase, 4CL 4-coumaroyl-CoA ligase, CHS chalcone synthase, CHI chalcone isomerase, F3′H flavonoid 3′-hydroxylase

In plants, the eriodictyol biosynthetic pathway starts from L-tyrosine, the general precursor for flavonoid biosynthesis (Fig. 1A). Under the action of tyrosine ammonia-lyase (TAL) and 4-coumarate-CoA ligase (4CL), L-tyrosine is converted to coumaroyl-CoA, which then forms naringenin chalcone by chelating malonyl-CoA at a 1:3 molar ratio via chalcone synthase (CHS). Then, naringenin chalcone is isomerized into naringenin, an important precursor of eriodictyol, via chalcone isomerase (CHI). Finally, naringenin is converted to eriodictyol by flavonoid 3′-hydroxylase (F3′H), which is a membrane-bound cytochrome P450 monooxygenase [7, 8]. The eriodictyol biosynthetic pathway, when introduced into bacteria, is usually inefficient due to the difficulty in heterologous expression of P450 enzymes. Functional expression of plant-derived P450 enzymes often requires various modifications, such as removal of the signal peptides or membrane-binding regions, and selection of appropriate P450 reductases [9–11]. An alternative is screening and selection of suitable non-P450 enzymes for the hydroxylation reactions originally catalyzed by P450 enzymes. One such enzyme is HpaBC native to some strains of Gram-negative bacteria such as *Escherichia coli*, *Pseudomonas putida*, and *Klebsiella* species [12–14]. HpaBC is a 4-hydroxyphenylacetate 3-hydroxylase with a broad substrate range and is the first sequenced enzyme in the family of two-component aromatic hydroxylases [13, 14]. This enzyme contains a 4-hydroxyphenylacetate 3-monooxygenase (HpaB) and a NAD(P)H-flavin oxidoreductase (HpaC) [15, 16]. HpaB alone has very low hydroxylase activity unless HpaC is present [13, 16]. HpaBC overexpressed episomally in recombinant *E. coli* has been utilized to catalyze *ortho*-hydroxylation reactions of a wide range of plant phenolic compounds such as flavonoids and stilbenes [12, 17–21].

The earliest attempts of microbial production of eriodictyol were achieved in *S. cerevisiae* and *E. coli* from caffeic acid with a respective titer of 6.5 mg/L and ~ 52 mg/L [22–25]. In all these pathways, the selection of precursors bypassed the step of P450-catalyzed hydroxylation. Later efforts have explored the conversion from tyrosine or naringenin still in *E. coli* and *S. cerevisiae*, either using *E. coli*-based HpaBC [17, 18] or via functionally expressed F3’H and P450 reductase identified by screening [26, 27] or engineering [28, 29]. These studies resulted in eriodictyol production at 13–200 mg/L. Recently, high-level production of eriodictyol was achieved in *S. cerevisiae* through expression of a F3′H and a cytochrome P450 reductase from *Silybum marianum*. Combined with promoter optimization and directed evolution of these enzymes, 3.3 g/L of eriodictyol was obtained from naringenin with a conversion rate of 62%, representing the highest titer reported to date [7].

In the past decade, *Corynebacterium glutamicum* has been attracting some attention since it is a GRAS (generally regarded as safe) bacterium not easily attacked by bacteriophages and is widely used in industry for the production of amino acids such as L-glutamate and L-lysine. Recently, this bacterium has been engineered to produce several subgroups of flavonoids and stilbenes, including flavanones, flavonols, pterostilbenes, and anthocyanins [30–32]. In addition, production of eriodictyol has also been reported in *C. glutamicum* using caffeic acid as a precursor with a final titer of 37 mg/L [32]. These studies demonstrate the applicability of *C. glutamicum* for the biosynthesis of various plant natural products.

In this study, we constructed a recombinant *C. glutamicum* strain capable of producing eriodictyol from tyrosine. Through screening of gene orthologues encoding pathway enzymes, enhancement in the supply of the precursor malonyl-CoA, introduction of *hpaBC* from *E. coli*, and optimization of fermentation conditions, the engineered strain was able to produce 14.10 mg/L of eriodictyol from tyrosine. To the best of our knowledge, this is the first report of *C. glutamicum*-based eriodictyol biosynthesis from tyrosine, and this study further broadens the potential of *C. glutamicum* for the production of hydroxylated flavonoids.

## Results

### **Construction of naringenin biosynthetic module in*C. glutamicum***

**Fig. 2:**
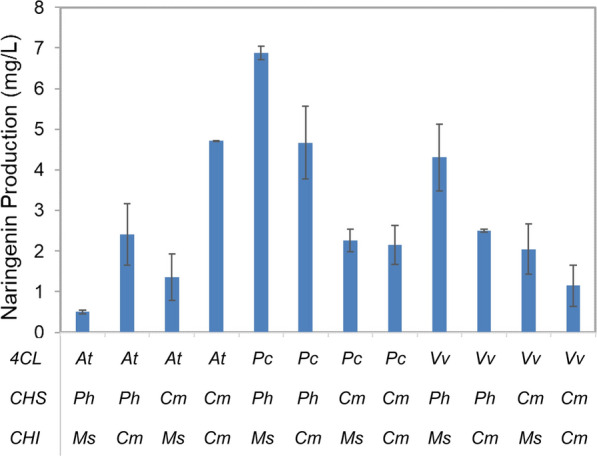
Naringenin production by recombinant *C. glutamicum* strains harboring 12 different combinations of *4CL*, *CHS* and *CHI* orthologues. Abbreviations: ***At***, *Arabidopsis thaliana*; ***Pc***, *Petroselinum crispum*; ***Vv***, *Vitis vinifera*; ***Ph***, *Petunia hybrida*; ***Cm***, *Citrus maxima*; ***Ms***, *Medicago sativa*. Data represents mean ± standard deviation of three independent experiments

Eriodictyol can be produced from naringenin in a single step. Therefore, to produce eriodictyol from tyrosine, we first introduced into *C. glutamicum* the naringenin biosynthetic pathway consisting of *TAL*, *4CL*, *CHS*, and *CHI* (Fig. 1 A), all of which were expressed under the T7 promoter in a monocistronic form (Fig. 1B). *TAL* from *Rhodotorula glutinis* was chosen due to the high activity and strong preference of the TAL enzyme for tyrosine over phenylalanine [33]. The other three genes were selected from various sources and tested for the catalytic efficiency of the relevant enzymes. Specifically, *4CL* was from *Arabidopsis thaliana* (*At*), *Petroselium crispum* (*Pc*), and *Vitis vinifera* (*Vv*); *CHS* was derived from *Petunia hybrida* (*Ph*) or *Citrus maxima* (*Cm*), whereas *CHI* had an origin of *Cm* or *Medicago sativa* (*Ms*). Sequence alignment of the corresponding enzyme orthologues showed 64–69% identity (78–84% similarity) between At4CL (NCBI accession number QJD21997.1), Pc4CL (CAA31696.1) and Vv4CL (AEX32786.1), 86% identity (92% similarity) between PhCHS (CAA27718.1) and CmCHS (ACX37403.1), and 54% identity (72% similarity) between MsCHI (AAB41524.1) and CmCHI (ADB92596.1). Assembly of these orthologues generated 12 recombinant pathway modules, which were then assayed for their capabilities of directing naringenin biosynthesis in shake flasks in AMM medium [30] supplemented with tyrosine. The titers of naringenin at different time points were determined, and the highest from each module was recorded and compared. Different combinations of orthologues exhibited more than 10-fold variations in naringenin production (0.50–6.98 mg/L), with *Pc4CL*-containing modules constantly yielding more naringenin than the *Vv4CL* counterparts regardless of the sources of *CHS* and *CHI* (Fig. 2). For *At4CL*, and for gene orthologues of *CHS* and *CHI*, it appeared that the performance of the pathway modules was independent of the origin of any individual genes but rather related to gene combinations. In addition, the CHS orthologues, which showed the highest degree of sequence identity and similarity among all the alignments, still led to large variations in the titer of naringenin. Among all the modules, the combination of *Pc4CL*, *PhCHS* and *MsCHI* resulted in the highest naringenin production, and was hence used in subsequent studies.

### Enhancement in the supply of malonyl-CoA for better naringenin production

**Fig. 3:**
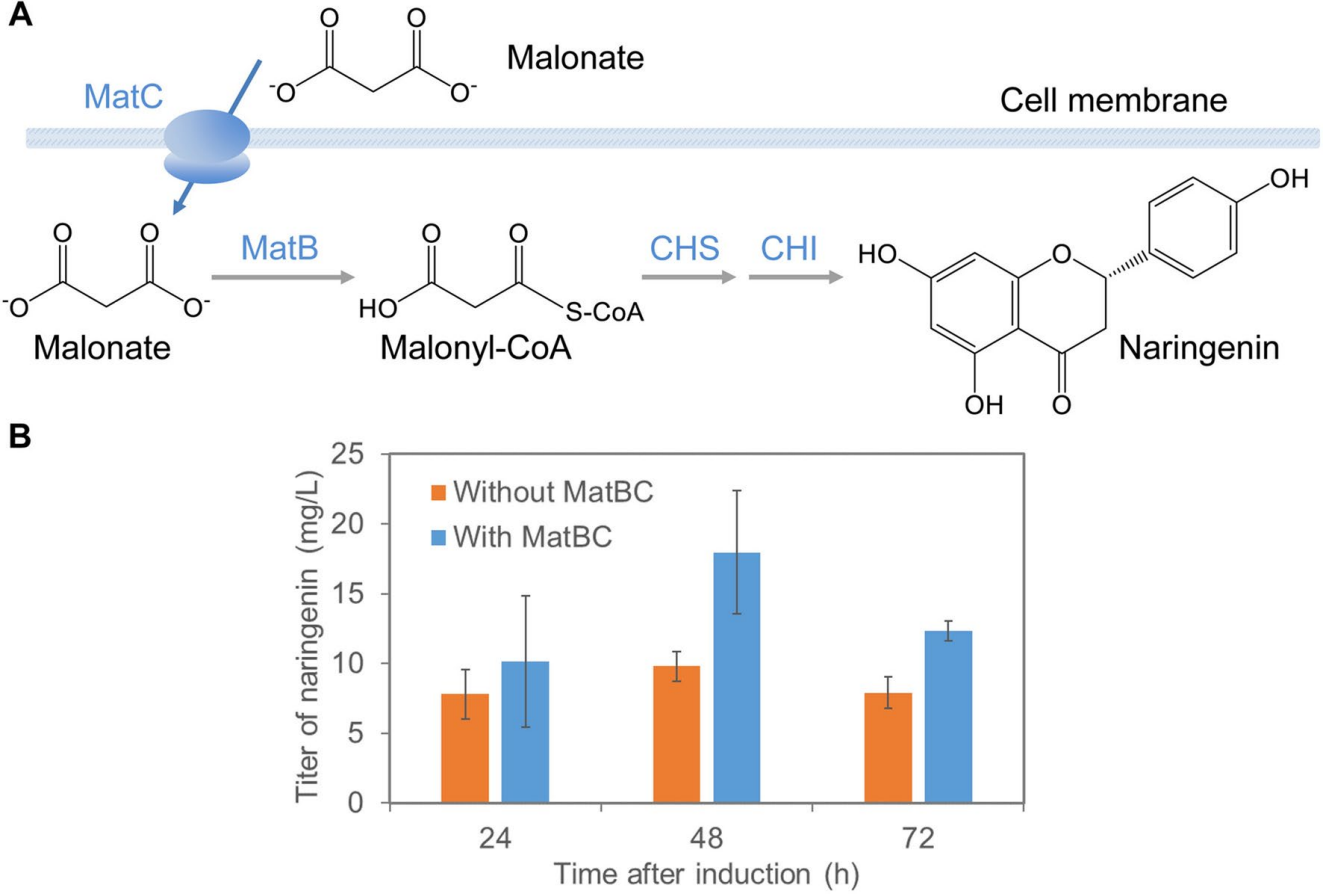
Introduction of malonate-to-malonyl-CoA pathway to increase naringenin production. (A) The malonyl-CoA synthetic pathway from malonate. Malonate is transported into cells by the MatC transporter and converted to malonyl-CoA by MatB. (B) The time-dependent naringenin production by recombinant *C. glutamicum* strains with (carrying plasmid pEKEx3-MatBC) or without (carrying plasmid pEKEx3) the expression of MatBC when fed with sodium malonate. Data represents mean ± standard deviation of three independent experiments

Malonyl-CoA is a co-substrate of CHS (Fig. 1), and its abundance is critical to naringenin production [19, 24]. As an important metabolic intermediate involved in fatty acid biosynthesis, malonyl-CoA is generally of low availability, restricting the high-level production of flavonoids [34]. To provide sufficient amount of malonyl-CoA for naringenin biosynthesis without greatly disturbing its metabolic pathway, we introduced a heterologous pathway, which is capable of converting malonate to malonyl-CoA, into *C. glutamicum* harboring the naringenin biosynthetic pathway (Fig. 3 A). This malonyl-CoA pathway consists of the malonate transporter gene *matC* and the malonyl-CoA synthase gene *matB* derived from *R. trifolii* [24]. These genes were expressed on plasmid pEKEx3-MatBC under the control of the *tac* promoter. We then tracked naringenin production at 24, 48, and 72 h of fermentation in the engineered *C. glutamicum* strains by feeding 2 g/L of sodium malonate into the growth medium in shake flasks. We observed an increase in the titer of naringenin at all time points compared with the strain expressing the naringenin pathway alone. The highest titer (17.96 mg/L) was reached after 48 h of fermentation, representing 83% improvement over the strain without MatBC (Fig. 3B). Naringenin accumulation declined at 72 h of fermentation in strains either with or without MatBC, suggesting possible degradation [32]. It is interesting to note that although naringenin production was time-dependent in both strains, the time-to-time difference was more prominent in the strain expressing MatBC.

### **Construction of the synthesis module of eriodictyol in*C. glutamicum***

**Fig. 4:**
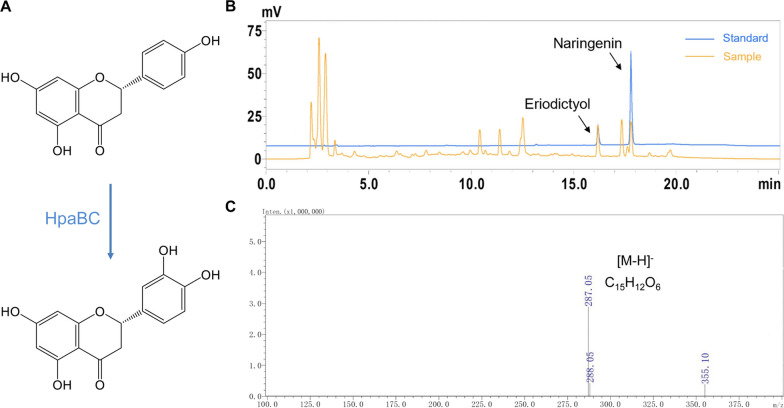
Eriodictyol production from naringenin via the expression of HpaBC in naringenin-producing *C. glutamicum*. **A** The hydroxylation reaction of naringenin conversion to eriodictyol catalyzed by HpaBC. **B** HPLC chromatograms of the naringenin and eriodictyol standards and the compounds produced from the recombinant strain expressing HpaBC. **C** Mass spectrometry analysis of eriodictyol in the fermentation products of recombinant *C. glutamicum* strains

To convert naringenin to eriodictyol using a non-P450 hydroxylase, we introduced HpaBC from *E. coli* to catalyze the hydroxylation reaction [18] (Fig. 4 A). The genes *hpaB* and *hpaC* were cloned from the genomic DNA of *E. coli*, assembled in monocistronic form under the control of promoter *Ptac* on plasmid pEKEx3-MatBC downstream of *matBC*, and introduced into naringenin-producing *C. glutamicum*. The metabolic performance of this strain was assayed in medium supplemented with sodium malonate and tyrosine in shake flasks. At 24 h of fermentation post induction with 0.5 mM IPTG, eriodictyol was detected in the fermentation culture with HpaBC expression as confirmed by LC-MS analysis (Fig. 4B and C). Therefore, it can be concluded that HpaBC from *E. coli* is functional in *C. glutamicum* and can convert naringenin to eriodictyol. However, only a small portion of naringenin was converted, indicating the necessity of further process optimization.

### Optimization of fermentation conditions for eriodictyol production

**Fig. 5:**
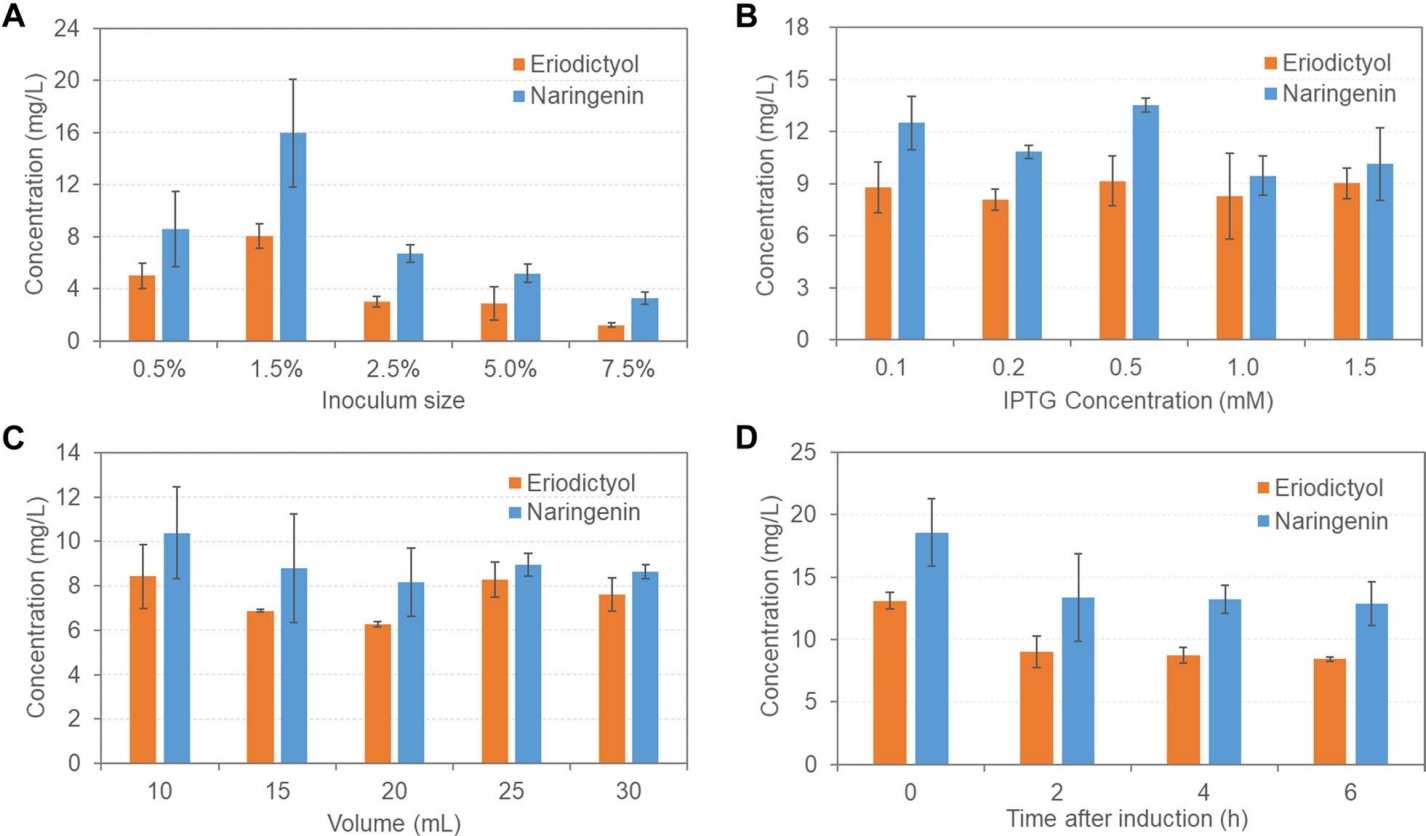
Optimization of fermentation conditions for improved eriodictyol production. The optimized bioprocess parameters include (**A**) inoculum size, (**B**) IPTG concentration, (**C**) fermentation volume, and (**D**) time of supplementation of malonate and tyrosine after IPTG induction. Data represents mean ± standard deviation of three independent experiments

To increase the conversion rate of naringenin and the titer of eriodictyol, we optimized several crucial fermentation conditions, including inoculum size, dissolved oxygen, IPTG concentration, carbon source, nitrogen source, and time of tyrosine addition (substrate delay). First, the overnight seed culture was inoculated into AMM medium at different ratios ranging from 0.5 to 7.5%. As shown in Fig. 5 A, the highest production of either naringenin or eriodictyol was achieved with 1.5% inoculum size. Higher inoculation ratio led to sharp decline in the titers of both compounds.

Next, the effect of IPTG concentration (ranging from 0.1 to 1.5 mM) on the production process was studied using 1.5% inoculation ratio. The production of both naringenin and eriodictyol was not very sensitive to IPTG concentration, and the optimum production was obtained with 0.5 mM IPTG induction (Fig. 5B), which was then adopted for all the subsequent optimization studies.

To optimize the level of dissolved oxygen in the fermentation system, we used an indirect method by changing the volume of fermentation culture from 10 to 30 mL in 100 mL shake flasks while keeping the agitation speed constant. A lower volume corresponds to more vigorous agitation and more supply of oxygen. The effect of culture volume on the production of naringenin and eriodictyol followed a similar nonlinear trend, and the highest titers of naringenin and eriodictyol were obtained with 10 mL of fermentation volume (Fig. 5C).

Previous literature has reported that the presence of flavonoid compounds may cause cell damage [18]. To minimize the possible negative effect, we intended to delay the production of naringenin and eriodictyol by adding the substrate tyrosine at different time points after IPTG induction of pathway enzyme expression. The highest titers of both naringenin and eriodictyol were achieved when tyrosine and IPTG were added simultaneously (Fig. 5D), probably due to high cell tolerance to the concentrations of both compounds produced.

**Fig. 6:**
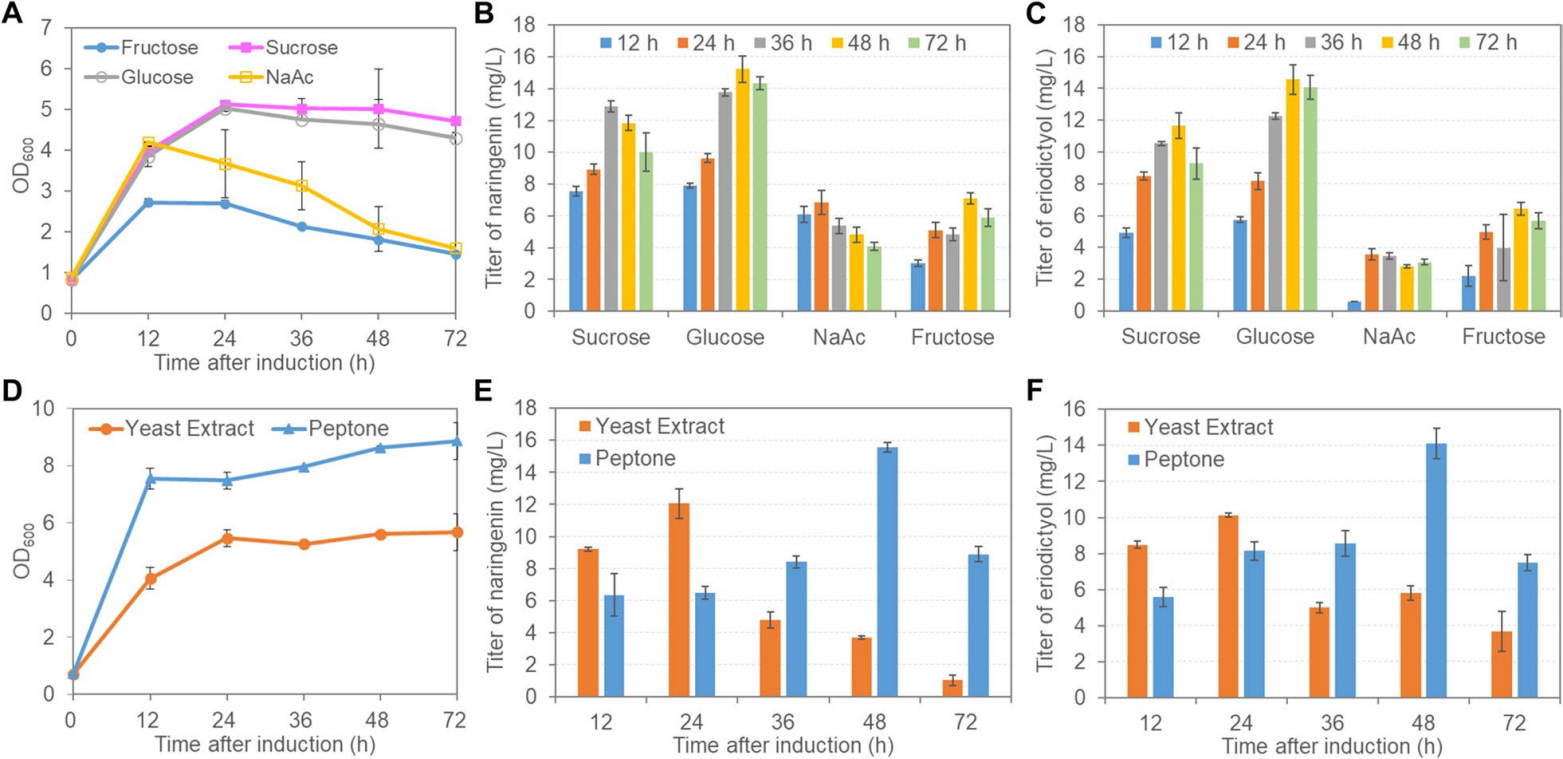
Effect of different carbon sources (**A**–**C**) and nitrogen sources (**D**–**F**) on (**A**, **D**) cell growth, (**B**, **E**) naringenin production, and (**C**, **F**) eriodictyol production. Data represents mean ± standard deviation of three independent experiments

We further investigated the impact of carbon and nitrogen sources on cell growth and eriodictyol production. Among the four carbon sources tested, sucrose and glucose supported fastest cell growth (Fig. 6A), while glucose resulted in the highest concentrations of naringenin and eriodictyol at almost all the time points peaking at 48 h post induction (Fig. 6B and C). The nitrogen sources tested were 2 g/L of peptone or yeast extract, and faster cell growth was constantly observed in peptone (Fig. 6D). The titers of naringenin and eriodictyol were both higher within 24 h when yeast extract was used; however, naringenin appeared to be degraded rapidly thereafter leading to inefficient production of eriodictyol (Fig. 6E and F). In contrast, peptone supported slow yet steady accumulation of naringenin and eriodictyol, with the highest eriodictyol titer of 14.10 mg/L obtained at 48 h post induction.

In summary, the optimum fermentation condition was set as 1.5% inoculation size in 10 mL culture using glucose as the carbon source and peptone as the nitrogen source, and enzyme expression was induced with 0.5 mM IPTG at 7 h of fermentation, at which time point the substrate tyrosine was supplemented. Such optimization improved the production of eriodictyol in *C. glutamicum* by one-fold compared with the production from the un-optimized fermentation system.

## Discussion

*C. glutamicum* is a promising strain to produce diverse flavonoids and has attracted much attention in recent years. In this study, we constructed a recombinant *C. glutamicum* that can produce eriodictyol from tyrosine. Through pathway engineering via selection of gene orthologues, enhancement of malonyl-CoA supply, and expression of the non-P450 hydroxylase HpaBC, and by means of process optimization, the eriodictyol production reached 14.10 mg/L. This study demonstrates the potential of *C. glutamicum* in the production of eriodictyol and other flavonoids for future applications, which could be coupled with tyrosine- or phenylalanine-producing *C. glutamicum* strains in a co-culture system.

In the current study, we applied HpaBC from *E. coli* to conduct the hydroxylation at 3’ position in the B ring of naringenin, which is the first attempt in *C. glutamicum*. In previous eriodictyol production using *C. glutamicum* as a host, the production process lies in the supplementation of caffeic acid, in which the C3’ site is already replaced by a hydroxyl group [32]. In other microbial production systems, the hydroxylation is generally carried out by the introduction of P450 hydroxylase. In *E. coli*, the expression of P450 hydroxylase, such as F3′-hydroxylase from plants, requires the removal of hydrophobic membrane anchors to maintain the activity and selection of appropriate cytochrome P450 reductase. In contrast, the expression of HpaBC, a native enzyme in *E. coli*, is much more convenient than the expression of P450 enzyme in *E. coli* and other prokaryotic microorganisms, such as *C. glutamicum* in this study. Expression of HpaBC does not need any special enzyme engineering efforts. Additionally, HpaBC has a spectrum of substrates to be hydroxylated at ortho-position, resulting in diverse flavonoid compounds [17]. Among them, naringenin is the most preferred substrate. In the current study, the highest conversion yield of naringenin to eriodictyol is about 50%, equivalent to other studies [7, 17, 18].

The enzyme complex HpaBC generally has a great demand for oxygen in the hydroxylation reaction [20]. However, we found that eriodictyol production was not linearly correlated with the culture volume, which is an indirect measure of oxygen supply. The highest eriodictyol production was actually achieved when the culture volume was either 10 mL or 25 mL, and the titer of eriodictyol was higher in 25-mL and 30-mL systems than in 15-mL and 20-mL culture. This was unexpected due to less oxygen supply in larger fermentation volumes. Given the same concentrations of medium components used for various culture volumes, a possible explanation is that cells cultured in larger volumes could accumulate more biomass and produce more eriodictyol due to the presence of more nutrients, which could partially compensate for the lower specific yield of eriodictyol produced by each single cell.

The limited supply of malonyl-CoA has been recognized as a bottleneck in the biosynthesis of flavonoids in microbial hosts such as *C. glutamicum* and *E. coli* [35]. Since malonyl-CoA is closely related to the endogenous biosynthesis of fatty acids, strategies that aim to inhibit fatty acid synthesis have been adopted with much success. These include the supplementation of the antibiotic cerulenin that blocks the activity of FabB and FabF in fatty acid synthesis [24, 32], the overexpression of acetyl-CoA carboxylase AccBC and AccD1 [34], and deletion of the gene encoding FasR that represses the transcription of *accBC* and *accD1* [34, 36]. However, FasR in *C. glutamicum* also represses the expression of fatty acid synthase Fas-IA and Fas-IB [37]. Thus, the deletion of *fasR* has a dual role to increase both the synthesis and consumption of malonyl-CoA, making it difficult to predict the actual change in the abundance of malonyl-CoA. Other strategies for improving the pool of malonyl-CoA involves modifications of the central metabolic pathway to increase the availability of acetyl-CoA, which include enhancement of the uptake of carbon sources, increase in the flux towards glycolysis, and partial elimination of the anaplerotic pyruvate carboxylation, etc. [34, 35].

Regarding the pathway enzyme orthologues, the best combination of 4CL, CHS and CHI orthologues in *C. glutamicum* is different from that in *E. coli* [38] although the enzyme sequences and promoters used are the same for the two systems. In the *E. coli*-based system, 4CL was identified as the key enzyme to determine the final production titer, and At4CL always led to higher naringenin production irrespective of the sources of CHS and CHI [38]. In the current study using the *C. glutamicum*-based system, although the decisive role of 4CL was not observed, its origin (more specifically, the protein sequence) was more critical than that of the other two enzymes in determining the final production titer. This is consistent with previous findings that 4CL catalyzes the conversion of *p*-coumaric acid to 4-coumaryol-CoA, which is considered as a difficult and rate-limiting reaction [39, 40]. However, the detailed reasoning behind the importance of the origin of 4CL is not clear, which could be due to poor expression or slow kinetics of this enzyme, instability or short half-life of 4-coumaryol-CoA, or strict selectivity of the enzyme. Moreover, high sequence similarity among any single set of enzyme orthologues, especially CHS, did not translate into similar naringenin production titers, indicating that it is the enzyme combination rather than a particular enzyme sequence that determines the catalytic efficiency of the pathway. This further suggests the significant role of compatibility of the enzymes along a pathway, likely on both the expression level and the catalytic level. More studies are needed to explore these complicated issues so as to better guide microbial synthesis of flavonoids and other plant natural products.

## Conclusions

We have demonstrated successful production of eriodictyol in *C. glutamicum* from tyrosine, which is more abundant and cheaper than previously used precursors naringenin and caffeic acid. Through screening of pathway gene orthologues and introduction of MatBC to increase the intracellular supply of malonyl-CoA, we achieved naringenin production from tyrosine in the first step. Further expression of *E. coli*-derived HpaBC followed by process optimization enabled 14.10 mg/L of eriodictyol produced from tyrosine. This is the first report on eriodictyol biosynthesis from tyrosine in *C. glutamicum*, and opens up new possibilities of microbial synthesis of plant flavonoids in this GRAS microbe. Since high-titer synthesis of tyrosine from glucose has been achieved in *C. glutamicum* [41, 42], it can be anticipated that *de novo* biosynthesis of eriodictyol from cheap carbon sources using *C. glutamicum* mono-culture or multi-cultures can be achieved.

## Methods

### Bacterial strains and media

*E. coli* DH5α was used for cloning and plasmid propagation and was grown in Luria Broth (LB) medium supplemented with 50 mg/L kanamycin or spectinomycin when necessary; agar was added to 20 g/L for the preparation of agar plates. *C. glutamicum* ATCC 13032 (DE3) was used as the host for flavonoid production in this study. *C. glutamicum* was generally grown in Brain Heart Infusion (BHI) medium and kept in BHI with glycerol (20%, v/v) at −80 ℃ for long-term storage. Fermentation by *C. glutamicum* was conducted at 30 ℃ in modified AMM medium [30] containing 0.2 mg/L of biotin with casamino acids replaced by peptone.

### Plasmid and strain construction

To construct the plasmids expressing *TAL*, *4CL*, *CHS* and *CHI*, the fragments containing *4CL*, *CHS* and *CHI* were released from previously constructed plasmids [38] by *Apa*I and *Nhe*I and ligated into the *Apa*I-*Avr*II digested plasmid pETM6-RgTAL [19]. The four genes were then excised and cloned into the *C. glutamicum*-*E. coli* shuttle vector pZM1 [30] by *Apa*I and *Nhe*I, forming the plasmids used for this study.

To construct the plasmid for the expression of the malonate-to-malonyl-CoA pathway, genes *matB* and *matC* from *Rhizobium trifolii* were PCR amplified using the plasmid pACYC-MatBC [24] as the template with primers MatB-F-BamHI/MatB-R and MatC-F/MatC-R-MfeI, and assembled together by overlap extension PCR. The fragment was then digested by *Bam*HI and *Mfe*I and cloned into *Bam*HI/*Eco*RI-digested pEKEx3, forming pEKEx3-MatBC.

To construct the plasmid carrying the eriodictyol biosynthesis module, the *HpaB* and *HpaC* genes were amplified from the genomic DNA of BL21(DE3) using primers HpaB-NdeI-F/ HpaB-SpeI-R and HpaC-NdeI-F/HpaC-SpeI-R and were then separately inserted into the plasmid pZM1 digested with *Nde*I and *Spe*I. The expression cassette of HpaC, consisting of the promoter P*tac*, the gene *HpaC*, and the T7 terminator, was amplified with the primer pair pZM1-HpaC-F/R and inserted into pZM1-HpaB through in-fusion cloning (Takara Bio) to obtain the plasmid pZM1-HpaBC. Subsequently, the expression cassettes of HpaB and HpaC in pZM1-HpaBC were amplified by primer pair pZM1-HpaBC-F/R and cloned into plasmid pEKEx3-MatBC by in-fusion cloning using primers pEKEx3-MatBC-F1/F/R/R2, resulting in the plasmid pEKEx3-MatBC-HpaBC.

The plasmids were electroporated into *C. glutamicum* ATCC 13032 (DE3) using an established method [30]. Briefly, competent cells were thawed on ice, mixed with ~ 100 ng of each plasmid, and transferred to an electroporation cuvette (2 mm gap). Cells were electroporated at 25 µF, 200 W and 2.5 kV using a Bio-Rad electroporator, and mixed with 1 mL pre-warmed BHI, followed by heat shock at 46 °C for 6 min. Cells were recovered at 30 °C for 2 h, and spread onto LB-agar plates containing antibiotics when necessary. Positive clones were selected by colony PCR, and plasmids were confirmed by sequencing (Sangon, Shanghai, China).

The plasmids and primers used for the cloning work are listed in Tables 1 and 2, respectively.

**Table 1:** Strains and plasmids used in this study

Plasmids or strains	Relevant properties	Source or references
*Escherichia coli* DH5α	Cloning of plasmids	Invitrogen
*E. coli* BL21 Star™ (DE3)	Template for cloning of HpaB and HpaC	Invitrogen
*C. glutamicum* (DE3)	Host, integrating T7 RNA polymerase	[43]
pZM1	An *E. coli*-*C. glutamicum* shuttle vector, pHM1519 ori, Kan^R^	[30]
pEKEx3	An *E. coli*-*C. glutamicum* shuttle vector, pBL1 ori, Spec^R^	[32]
pETM6-RgTAL	pETM6 carrying *R. glutinis TAL*	[19]
pZM1-HpaB	pZM1 carrying HpaB from *E. coli*	This study
pZM1-HpaC	pZM1 carrying HpaC from *E. coli*	This study
pZM1-HpaBC	pZM1 carrying HpaB and HpaC from *E. coli*	This study
pZM1-RgTAL-x4CL-yCHS-zCHI	pZM1 carrying *R. glutinis TAL, 4CL, CHS*, and *CHI.* X means genes from *Arabidopsis thaliana*, *Petroselinum crispum*, and *Vitis vinifera*; y means genes from *Petunia hybrida* and *Citrus maxima* (Cm); z means gene from Cm and *Medicago sativa*.	This study
pACYC-MatBC	pACYCDuet-1 carrying *R. trifolii* MatB and MatC	[24]
pEKEx3-MatB	pEKEx3 carrying *R. trifolii* MatB	This study
pEKEx3-MatBC	pEKEx3 carrying *R. trifolii* MatB and MatC	This study
pEKEx3-MatBC-HpaBC	pEKEx3 carrying *R. trifolii* MatB and MatC, and *E. coli* HpaB and HpaC	This study

**Table 2:** Oligonucleotides used in this study

Primers	Sequence (5’–3’ with restriction sites underlined)
HpaB-NdeI-F	GGGCCTCATATGAAACCAGAAGATTTCCGCGCCAG
HpaB-SpeI-R	GGGCCCACTAGTTATTTCAGCAGCTTATCCAGCATGTTGA
HpaC-NdeI- F	GGGTTTCATATGCAATTAGATGAACAACGCCTGCGC
HpaC-SpeI-R	GGGCCCACTAGTTAAATCGCAGCTTCCATTTCCAGCATCA
pZM1-HpaC-F	CTAGCGAAAGGAGGAGTTGACAATTAATCATCGGCTCGTATAATGTGTGGA
pZM1-HpaC-R	AGAGTTTGTAGAAACGCGTCGACTCCTC
MatB-F-BamHI	CCGGGATCCAAAGGAGGACAACCATGAGCAACCATCTTTTCGACG
MatB-R	CGACCTCCTTTAGCACCTTACGTCCTGGTATAAAGATCGGC
MatC-F	CGTAAGGTGCTAAAGGAGGTCGAAGATGGGTATTGAATTACTGTCCATAGG
MatC-R-MfeI	CCGCAATTGTCAAACCAGCCCGGGC
pEKEx3-MatBC-F1	AGCGCATTGTTAGATTTCATACACGG
pEKEx3-MatBC-F	CAGGAACCGTAAAAAGGCCGCGTTG
pEKEx3-MatBC-R	TTGTAGAAACGCAAAAAGGCCATCC
pEKEx3-MatBC-R2	TGAAATCTAACAATGCGCTCATCGTCA
pZM1-HpaBC-F	CGGCCTTTTTACGGTTCCTGTTGTAGAAACGCGTCGACTCCTCC
pZM1-HpaBC-R	GCCTTTTTGCGTTTCTACAATCCCAGTCACGACCCTAGGG

### Fermentation conditions

Glycerol stocks were streaked onto LB-agar plates with 25 mg/L kanamycin and 100 mg/L spectinomycin. Single colonies were inoculated into 3 mL of BHI medium with 25 mg/L kanamycin and 100 mg/L spectinomycin in a 20-mL culture tube for overnight growth at 30 ℃ and 200 rpm. Fresh modified AMM (15 mL) with 25 mg/L kanamycin and 100 mg/L spectinomycin in a 100 mL flask was inoculated with 375 µL of the overnight culture, or other volumes when noted. After 7 h of cultivation at 30 ℃ and 200 rpm, IPTG was added to a final concentration of 0.5 mM or specified otherwise, and sodium malonate (prepared as a 100 g/L stock solution in deionized water) and tyrosine (prepared as a 25 mg/mL stock solution in DMSO) were also added to final concentrations of 2 g/L and 500 mg/L, respectively. For the optimization of carbon and nitrogen sources, the glucose in modified AMM was replaced with 20 g/L of fructose or sucrose, and peptone was replaced with 2 g/L yeast extract. The culture was further grown at 30 ℃ and 200 rpm for 72 h. Every 12 h, the optical density at 600 nm (OD_600_) was measured, and 200 µL of the culture were collected and stored at − 20℃ for later use.

### Metabolite analysis

The collected fermentation culture was mixed well with an equal volume of anhydrous ethanol, and centrifuged at 21,000×*g* for 10 min. The supernatants were analyzed by a previously established method [18]. Briefly, 10 µL of each sample was loaded into a Shimadzu Essentia LC-16 HPLC system equipped with a Unitary C18 column (5 μm, 4.6 × 250 mm) and a diode array detector. The mobile phase was acetonitrile with 0.1% formic acid (solvent A) and 0.1% formic acid in water (solvent B). HPLC program was set as 10–40% A at 0–10 min and 40–60% A at 10–15 min with a constant flow rate of 1 mL/min. Absorbance at 280 nm was monitored.

### LC-MS analysis

Agilent 1200 series HPLC equipped with an Eclipse XDB-C18 column (5 μm, 150 × 4.6 mm) and an LTQ-ORBITRAP XL mass spectrometer was used. HPLC analysis was performed with solvent A (0.1% formic acid in water) and solvent B (0.1% formic acid in acetonitrile) at a flow rate of 250 µL/min with a linear gradient (5% B at 0–5 min, 5–45% B at 5–40 min, 45–90% B at 40–45 min, 90% B at 45–49.9 min, 90–5% B at 49.9–50 min, and 5% B at 50–60 min). Mass spectrometer was operated in a negative ion mode with 2-ppm mass accuracy. Mass spectra were acquired at a resolution of 60,000 in a detection range of M/Z 100–700. Acquisition parameters were set as follows: spray voltage 4.5 kV, capillary voltage 44 V, tube lens voltage 150 V, capillary temperature 250 °C, sheath flow rate 25, and auxiliary gas flow rate 5.

## Data Availability

The data supporting our findings can be found in the main paper and the supplementary file.

## References

[1] Deng Z, Hassan S, Rafiq M, Li H, He Y, Cai Y, Kang X, Liu Z, Yan T (2020). Pharmacological activity of eriodictyol: the major natural polyphenolic flavanone. Evidence-Based Complementary Altern Med.

[2] Islam A, Islam MS, Rahman MK, Uddin MN, Akanda MR (2020). The pharmacological and biological roles of eriodictyol. Arch Pharmacal Res.

[3] Lee E-R, Kim J-H, Kang Y-J, Cho S-G (2007). The anti-apoptotic and anti-oxidant effect of eriodictyol on UV-induced apoptosis in keratinocytes. Biol Pharm Bull.

[4] Zha J, Wu X, Koffas MAG (2020). Making brilliant colors by microorganisms. Curr Opin Biotechnol.

[5] Marsafari M, Samizadeh H, Rabiei B, Mehrabi A, Koffas M, Xu P (2020). Biotechnological production of flavonoids: an update on plant metabolic engineering, microbial host selection, and genetically encoded biosensors. Biotechnol J.

[6] Pandey RP, Parajuli P, Koffas MAG, Sohng JK (2016). Microbial production of natural and non-natural flavonoids: pathway engineering, directed evolution and systems/synthetic biology. Biotechnol Adv.

[7] Gao S, Xu X, Zeng W, Xu S, Lyv Y, Feng Y, Kai G, Zhou J, Chen J (2020). Efficient biosynthesis of (2*S*)-eriodictyol from (2*S*)-naringenin in *Saccharomyces cerevisiae* through a combination of promoter adjustment and directed evolution. ACS Synth Biol.

[8] Dunstan MS, Robinson CJ, Jervis AJ, Yan C, Carbonell P, Hollywood KA, Currin A, Swainston N, Feuvre RL, Micklefield J, Faulon J-L, Breitling R, Turner N, Takano E, Scrutton NS (2020). Engineering *Escherichia coli* towards *de novo* production of gatekeeper (2*S*)-flavanones: naringenin, pinocembrin, eriodictyol and homoeriodictyol. Synth Biol.

[9] Li J, Tian C, Xia Y, Mutanda I, Wang K, Wang Y (2019). Production of plant-specific flavones baicalein and scutellarein in an engineered *E. coli* from available phenylalanine and tyrosine. Metab Eng.

[10] Leonard E, Yan Y, Koffas MAG (2006). Functional expression of a P450 flavonoid hydroxylase for the biosynthesis of plant-specific hydroxylated flavonols in *Escherichia coli*. Metab Eng.

[11] Fowler ZL, Koffas MAG (2009). Biosynthesis and biotechnological production of flavanones: current state and perspectives. Appl Microbiol Biotechnol.

[12] Lin Y, Yan Y (2012). Biosynthesis of caffeic acid in *Escherichia coli* using its endogenous hydroxylase complex. Microb Cell Fact.

[13] Prieto MA, Garcia JL (1994). Molecular characterization of 4-hydroxyphenylacetate 3-hydroxylase of *Escherichia coli.* A two-protein component enzyme. J Biol Chem.

[14] Prieto MA, Perez-Aranda A, Garcia JL (1993). Characterization of an *Escherichia coli* aromatic hydroxylase with a broad substrate range. J Bacteriol.

[15] Xun L, Sandvik ER (2000). Characterization of 4-hydroxyphenylacetate 3-hydroxylase (HpaB) of *Escherichia coli* as a reduced flavin adenine dinucleotide-utilizing monooxygenase. Appl Environ Microbiol.

[16] Galán B, Díaz E, Prieto MA, García JL (2000). Functional analysis of the small component of the 4-hydroxyphenylacetate 3-monooxygenase of *Escherichia coli* W: a prototype of a new flavin:NAD(P)H reductase subfamily. J Bacteriol.

[17] Wang L, Ma X, Ruan H, Chen Y, Gao L, Lei T, Li Y, Gui L, Guo L, Xia T, Wang Y (2021). Optimization of the biosynthesis of B-ring ortho-hydroxylated flavonoids using the 4-hydroxyphenylacetate 3-hydroxylase complex (HpaBC) of *Escherichia coli*. Molecules.

[18] Jones JA, Collins SM, Vernacchio VR, Lachance DM, Koffas MAG (2016). Optimization of naringenin and *p*-coumaric acid hydroxylation using the native *E. coli* hydroxylase complex, HpaBC. Biotechnol Prog.

[19] Jones JA, Vernacchio VR, Collins SM, Shirke AN, Xiu Y, Englaender JA, Cress BF, McCutcheon CC, Linhardt RJ, Gross RA, Koffas MAG (2017). Complete biosynthesis of anthocyanins using *E. coli* polycultures. mBio.

[20] Furuya T, Sai M, Kino K (2016). Biocatalytic synthesis of 3,4,5,3’,5’-pentahydroxy-trans-stilbene from piceatannol by two-component flavin-dependent monooxygenase HpaBC. Biosci Biotechnol Biochem.

[21] Ni J, Tao F, Du H, Xu P (2015). Mimicking a natural pathway for *de novo* biosynthesis: natural vanillin production from accessible carbon sources. Sci Rep.

[22] Leonard E, Lim K-H, Saw P-N, Koffas MAG (2007). Engineering central metabolic pathways for high-level flavonoid production in *Escherichia coli*. Appl Environ Microbiol.

[23] Yan Y, Kohli A, Koffas MAG (2005). Biosynthesis of natural flavanones in *Saccharomyces cerevisiae*. Appl Environ Microbiol.

[24] Leonard E, Yan Y, Fowler ZL, Li Z, Lim CG, Lim KH, Koffas MA (2008). Strain improvement of recombinant *Escherichia coli* for efficient production of plant flavonoids. Mol Pharmaceutics.

[25] Fowler ZL, Gikandi WW, Koffas MAG (2009). Increased malonyl coenzyme A biosynthesis by tuning the *Escherichia coli* metabolic network and its application to flavanone production. Appl Environ Microbiol.

[26] Amor IL, Hehn A, Guedone E, Ghedira K, Engasser JM, Chekir-Ghedrira L, Ghoul M (2010). Biotransformation of naringenin to eriodictyol by *Saccharomyces cerevisiea* functionally expressing flavonoid 3’ hydroxylase. Nat Prod Commun.

[27] Kasai N, Ikushiro S, Hirosue S, Arisawa A, Ichinose H, Wariishi H, Ohta M, Sakaki T (2009). Enzymatic properties of cytochrome P450 catalyzing 3’-hydroxylation of naringenin from the white-rot fungus *Phanerochaete chrysosporium*. Biochem Biophys Res Commun.

[28] Zhu S, Wu J, Du G, Zhou J, Chen J (2014). Efficient synthesis of eriodictyol from L-tyrosine in *Escherichia coli*. Appl Environ Microbiol.

[29] Chu LL, Pandey RP, Jung N, Jung HJ, Kim E-H, Sohng JK (2016). Hydroxylation of diverse flavonoids by CYP450 BM3 variants: biosynthesis of eriodictyol from naringenin in whole cells and its biological activities. Microb Cell Fact.

[30] Zha J, Zang Y, Mattozzi M, Plassmeier J, Gupta M, Wu X, Clarkson S, Koffas MAG (2018). Metabolic engineering of *Corynebacterium glutamicum* for anthocyanin production. Microb Cell Fact.

[31] Kallscheuer N, Vogt M, Bott M, Marienhagen J (2017). Functional expression of plant-derived *O*-methyltransferase, flavanone 3-hydroxylase, and flavonol synthase in *Corynebacterium glutamicum* for production of pterostilbene, kaempferol, and quercetin. J Biotechnol.

[32] Kallscheuer N, Vogt M, Stenzel A, Gätgens J, Bott M, Marienhagen J (2016). Construction of a *Corynebacterium glutamicum* platform strain for the production of stilbenes and (2*S*)-flavanones. Metab Eng..

[33] Vannelli T, Wei Qi W, Sweigard J, Gatenby AA, Sariaslani FS (2007). Production of *p*-hydroxycinnamic acid from glucose in *Saccharomyces cerevisiae* and *Escherichia coli* by expression of heterologous genes from plants and fungi. Metab Eng.

[34] Milke L, Kallscheuer N, Kappelmann J, Marienhagen J (2019). Tailoring *Corynebacterium glutamicum* towards increased malonyl-CoA availability for efficient synthesis of the plant pentaketide noreugenin. Microb Cell Fact.

[35] Milke L, Marienhagen J (2020). Engineering intracellular malonyl-CoA availability in microbial hosts and its impact on polyketide and fatty acid synthesis. Appl Microbiol Biotechnol.

[36] Milke L, Ferreira P, Kallscheuer N, Braga A, Vogt M, Kappelmann J, Oliveira J, Silva AR, Rocha I, Bott M, Noack S, Faria N, Marienhagen J (2019). Modulation of the central carbon metabolism of *Corynebacterium glutamicum* improves malonyl-CoA availability and increases plant polyphenol synthesis. Biotechnol Bioeng.

[37] Nickel J, Irzik K, van Ooyen J, Eggeling L (2010). The TetR-type transcriptional regulator FasR of *Corynebacterium glutamicum* controls genes of lipid synthesis during growth on acetate. Mol Microbiol.

[38] Jones JA, Vernacchio VR, Sinkoe AL, Collins SM, Ibrahim MHA, Lachance DM, Hahn J, Koffas MAG (2016). Experimental and computational optimization of an *Escherichia coli* co-culture for the efficient production of flavonoids. Metab Eng..

[39] Chen X, Wang H, Li X, Ma K, Zhan Y, Zeng F (2019). Molecular cloning and functional analysis of 4-coumarate:CoA ligase 4 (4CL-like 1) from *Fraxinus mandshurica* and its role in abiotic stress tolerance and cell wall synthesis. BMC Plant Biol.

[40] Xiong D, Lu S, Wu J, Liang C, Wang W, Wang W, Jin J-M, Tang S-Y (2017). Improving key enzyme activity in phenylpropanoid pathway with a designed biosensor. Metab Eng.

[41] Lütke-Eversloh T, Santos CNS, Stephanopoulos G (2007). Perspectives of biotechnological production of L-tyrosine and its applications. Appl Microbiol Biotechnol.

[42] Ikeda M, Katsumata R (1992). Metabolic engineering to produce tyrosine or phenylalanine in a tryptophan-producing *Corynebacterium glutamicum* strain. Appl Environ Microbiol.

[43] Kortmann M, Kuhl V, Klaffl S, Bott M (2015). A chromosomally encoded T7 RNA polymerase-dependent gene expression system for *Corynebacterium glutamicum*: construction and comparative evaluation at the single-cell level. Microb Biotechnol.

